# Supplementary figures and images for: Whole-Genome Identification of Regulatory Function of CDPK Gene Families in Cold Stress Response for Prunus mume and Prunus mume var. Tortuosa
Source: Plants (Basel). 2023 Jul 4;12(13):2548. doi: 10.3390/plants12132548 (PMC10346478; doi:10.3390/plants12132548)

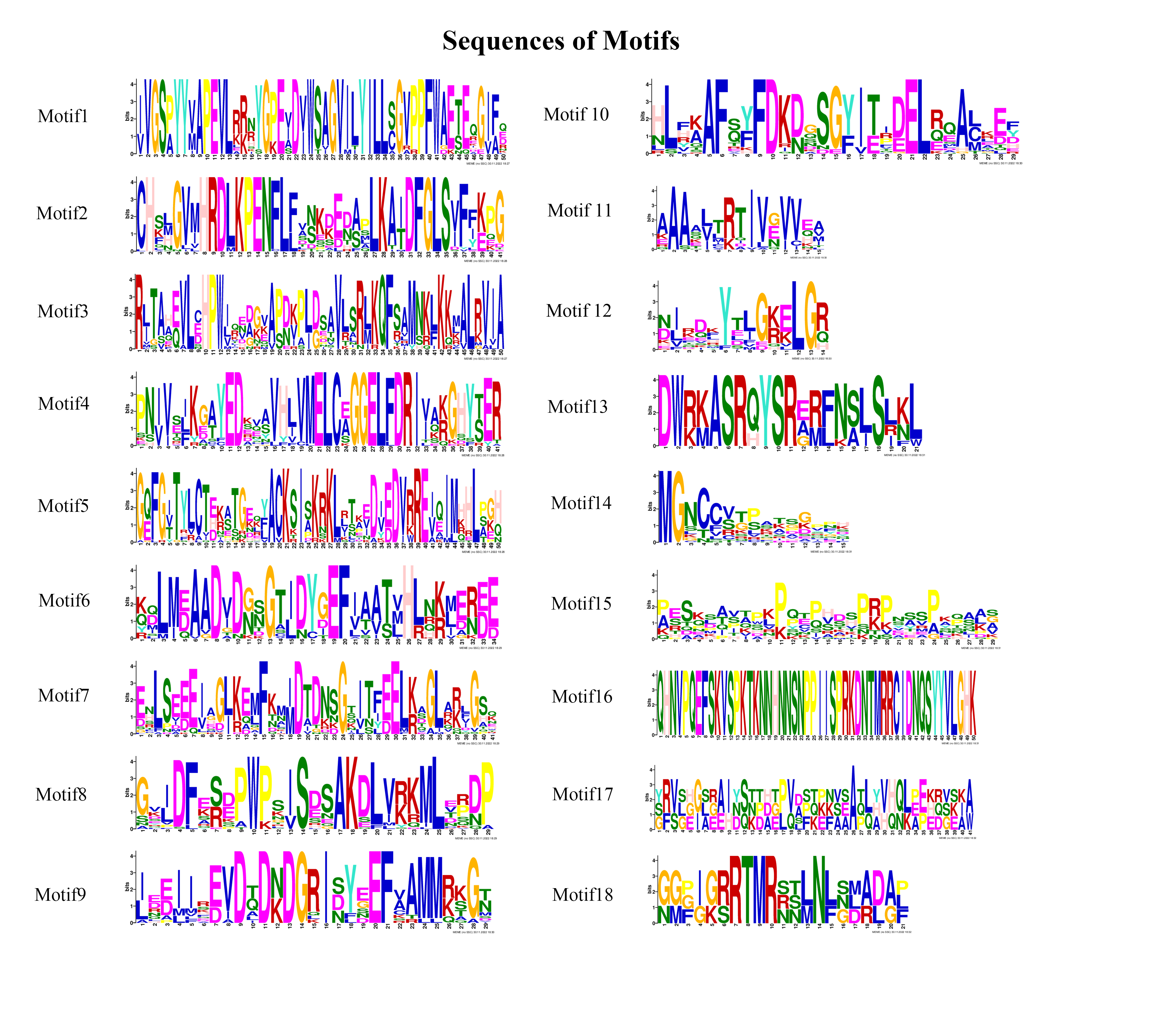

Supplement: Supplementary file 1 [file plants-12-02548-s001.zip › Figure S1.png]

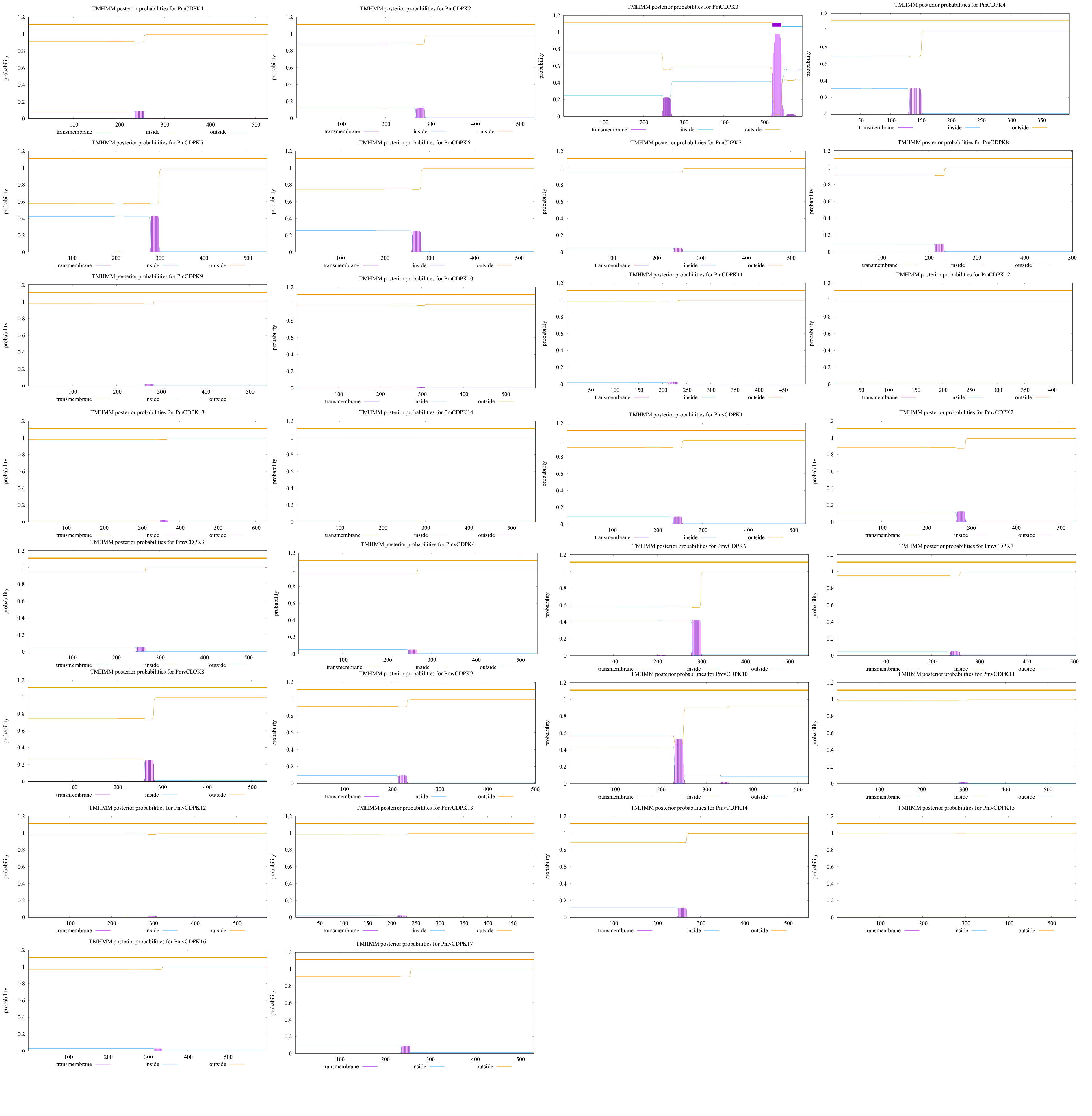

Supplement: Supplementary file 1 [file plants-12-02548-s001.zip › Figure S2.png]
